# Shear Bond Strengths of Composite Resin Bonded to MIH-Affected Hard Tissues with Different Adhesives and Pre-Treatments

**DOI:** 10.3390/dj13080377

**Published:** 2025-08-20

**Authors:** Cia Solanke, Hassan Shokoohi-Tabrizi, Andreas Schedle, Katrin Bekes

**Affiliations:** 1 Department of Paediatric Dentistry, University Clinic of Dentistry, Medical University of Vienna, Sensengasse 2a, 1090 Vienna, Austria; 2 Core Facility Applied Physics, Laser and CAD/CAM Technology, University Clinic of Dentistry, Medical University of Vienna, Sensengasse 2a, 1090 Vienna, Austria; 3 Competence Centre Dental Materials, University Clinic of Dentistry, Medical University of Vienna, Sensengasse 2a, 1090 Vienna, Austria

**Keywords:** molar incisor hypomineralisation, self-etch, etch-and-rinse, oxidative pre-treatment, resin infiltration

## Abstract

**Background**: Reduced bond strengths in hypmineralised enamel have been reported with increased restorative failures. This study aimed to investigate the shear bond strengths of resin composite to hypomineralised enamel and dentin bonded with two different adhesive systems and pre-treatments. **Methods**: Thirty-six freshly extracted first permanent molars with MIH and 17 sound third molars were used for shear bond strength tests in enamel and dentin. Specimens of control groups were bonded to resin composite using Scotchbond^TM^ Universal Plus and Adper^TM^ Scotchbond 1XT. MIH-affected enamel specimens of six test groups were pre-treated with various chemical agents, such as 35% phosphoric acid, 5% NaOCl, resin infiltration with ICON^®^, or a combination of these agents prior to bonding with composite resin using Scotchbond^TM^ Universal Plus. Bonded specimens were subsequently sheared at a crosshead speed of 1.0 mm/min, after which their fracture modes were recorded. The mean bond strengths of all groups were compared using a one-way analysis of variance test (ANOVA) and a Bonferroni–Holm analysis was performed for pairwise comparison between the groups. The association between modes of failure was examined with Pearson’s chi-square test. **Results**: Mean shear bond strength values were highest for sound dentin specimens (Group SD 2) bonded with Scotchbond^TM^ Universal Plus (23.76 ± 7.68 MPa). Sound enamel specimens (Group SE 2) exhibited significantly higher mean bond strength values than MIH-enamel specimens (Group HE 2) when bonded with Scotchbond^TM^ Universal Plus (19.68 ± 6.25 vs. 11.53 ± 3.29 MPa, *p* < 0.001). Oxidative pre-treatment followed by resin infiltration significantly improved bond strengths to hypomineralised enamel (Group HE 6) (17.84 ± 2.98 MPa, *p* < 0.05). Bond strengths to sound and hypomineralised enamel and dentin did not differ significantly for both adhesives. **Conclusions**: Within the limitations of an in vitro study, oxidative pre-treatment in combination with resin infiltration seems to be beneficial when planning adhesive restorations with composite in hypomineralised enamel. Both Scotchbond^TM^ Universal Plus and Adper^TM^ Scotchbond 1XT can be used for bonding of resin composite to MIH-affected enamel and dentin.

## 1. Introduction

Molar incisor hypomineralisation (MIH) is well-recognised as a qualitative enamel defect clinically characterised by well-demarcated opacities of varying colour, abnormal translucency, and reduced mineralisation. The phenomenon invariably affects one or more first permanent molars (FPMs), with the occasional involvement of permanent incisors [1]. However, more recently, similar hypomineralised enamel aberrations have also been reported in other teeth, such as permanent second molars, premolars, canines, primary canines (HPC), and primary second molars (HSPM) [2,3,4]. In fact, the presence of HSPM and/or HPC is a significant clinical factor that prognosticates the presence of MIH [5,6,7,8].

According to the most recent meta-analysis of 2018, a substantial 13.5% of the world’s population suffers from MIH [9,10]. Despite being globally prevalent and the long-standing research in the field of MIH aetiology, the exact cause of MIH continues to remain unknown. The latest aetiological evidence is suggestive of a multifactorial model with genetic and/or epigenetic factors synergistically influencing the amelogenesis of affected teeth [11].

Clinically, MIH-affected teeth exhibit great variability in their expression within patients as well as within an individual, ranging from hard, white chalky opacities to soft, porous, yellow-brown opacities associated with post-eruptive enamel breakdown and/or hypersensitivity [1]. As a result of this diverse clinical spectrum and associated factors such as impaired oral health-related quality of life of MIH patients, difficulty in achieving profound anaesthesia, and poor predictability of therapeutic and restorative outcomes, management of MIH is challenging for patients, caregivers, and dentists [12,13,14,15].

Recently, the treatment recommendations of MIH were updated by the Best Practice Clinical Guidelines of the European Academy of Paediatric Dentistry (EAPD) [16] as well as the Würzburg concept [17]. Treatment options mentioned in these guides range from prophylactic and restorative treatment to the extraction of severely affected MIH teeth. For the restorative treatment of MIH teeth using direct restorations with composite resin, two broad categories of treatments are available: invasive and non-invasive or minimally invasive treatments. While invasive restorative techniques advocate the complete removal of hypomineralised enamel (HE) and placement of cavo-surface angles in sound enamel tissue [18], non-invasive techniques identify with minimal intervention dentistry and are based on strengthening and preservation of hypomineralised enamel tissue [19]. Over the last decade an upsurge in the research of the latter has been observed due to the understanding that complete removal of hypomineralised tissue is associated with the risk of weakening already compromised crown structure and hence may not always be a viable treatment option and secondly due to the attainment of a comprehensive understanding of the structural, mechanical and chemical properties of hypomineralised hard tissues. Published reports from experiments investigating MIH-affected enamel have reported a reduction in mineral quantity and quality (reduced calcium and phosphate content), reduction of hardness and modulus of elasticity, an increase in porosity, carbon/carbonate concentrations, and protein content compared to unaffected enamel [20,21]. Non-invasive and minimally invasive restorative treatments aim at battling these abnormalities by altering, strengthening, and preserving hypomineralised tissue, thereby improving bonding to restorative materials.

Oxidative pre-treatment is a non-invasive treatment that uses deproteinising agents such as sodium hypochlorite (NaOCl) in varying concentrations to remove excess surface and inter-lesion protein in hypomineralised enamel. The eliminated excess protein, which has been suggested to act as a mechanical and chemical barrier, enhances the penetration of dental adhesives into hypomineralised enamel and improves the bond strength of resin composite to MIH enamel [22]. This pre-treatment method was initially investigated in enamel affected by hypocalcified amelogenesis imperfecta (HCAI), and promising results of increased bond strength to HCAI enamel encouraged its testing in hypomineralised enamel of MIH teeth [23]. Since then, several published in vitro studies have tested oxidative pre-treatment to HE with 5% NaOCl and reported mixed results pertaining bond strengths of composite resin and resin sealant [22,24,25,26,27]. Additionally, the use and non-use of acid etching post-oxidative treatment with NaOCl has also been investigated, and significant improvement in the bonding of resin sealants to HE with the prior method has been observed [26].

A minimally invasive treatment called resin infiltration has increasingly been researched for its effects in the aesthetic treatment of white spots [28], in the treatment of hypersensitive MIH teeth [29], and in improving the bonding of composite resin to hypomineralised hard tissue [22]. Resin infiltration uses a low viscosity triethyleneglycol dimethacrylate (TEGMA) resin with a high penetration coefficient to occlude highly porous structures of white spot lesions of enamel, inhibiting demineralisation by limiting diffusion of acid and by affording some mechanical support to the tissue [30]. In hypomineralised tissues, it was postulated to penetrate and seal porous HE, offer mechanical support within the tissue, and improve subsequent bonding of resin composite [22]. However, the high level of protein in HE was thought to act as a mechanical and chemical barrier and inhibit infiltration. As a result, it was suggested to combine oxidative pre-treatment with resin infiltration to help eliminate excessive proteins and enhance bond strengths to HE.

To the authors’ knowledge, only two published in vivo experiments compared the use of oxidative pre-treatment with and without resin infiltration, and both reported contradictory results [22,25]. Chay et al. reported increased micro-shear bond strength (MSBS) values to HE pre-treated with 5% NaOCl, with the use and non-use of ICON^®^ resin infiltration [22]. On the contrary, Kraemer et al. reported no difference in the micro-tensile bond strengths of composite resin to control and hypomineralised enamel of MIH-affected teeth using the same pre-treatments [25]. The results of these two experiments highlight the need for more investigations comparing the effects of multiple pre-treatments of MIH teeth and evaluating their effect on the bond strengths of composite resin. Furthermore, the evidence of the quality of published reports on non-invasive and minimally invasive treatments such as resin infiltration has been graded as “low” in the updated Best Clinical Practice Guidance [16], emphasising the need for high-calibre research in this field. It was therefore the aim of this study to compare the effect of different pre-treatments on shear bond strengths (SBSs) of composite resin to sound and hypomineralised hard tissues bonded with a universal dental adhesive in self-etch mode and an etch-and-rinse adhesive.

The null hypotheses tested were as follows: (1) There is no difference in shear bond strengths to enamel and dentin of sound and MIH-affected molars; (2) Various pre-treatments of HE do not influence adhesion to MIH enamel; and (3) There is no difference in shear bond strengths of resin composite between a universal adhesive in self-etch mode and an etch-and-rinse adhesive.

## 2. Methods

### 2.1. Teeth Collection and Study Sample

A total of 53 teeth, consisting of erupted first permanent molars (FPMs) affected with MIH (N = 36) and sound third molars (N = 17), were collected from paediatric and adult patients, respectively, who were undergoing treatment at the University Clinic of Dentistry in Vienna, Austria. Teeth were extracted due to medical reasons with informed consent approved by a the ethics committee of the Medical University of Vienna (Reg. Nr. #1065/2013, approval date: 12 March 2013).

Extracted teeth were temporarily stored in specimen bottles containing 4% buffered formaldehyde solution (Roti^®^-Histofix, Kalsruhe, Germany) for 2 weeks before they were cleaned using a wet, slow rotary brush to remove blood and soft tissue contaminants. The cleaned teeth were then rinsed with deionised distilled water (DDW) and inspected under daylight conditions by one investigator (KB). Clinical diagnosis of the MIH-affected molars was confirmed by using the judgement criteria described by Weerheijm et al. [31]. The inclusion criteria for sound and MIH-affected teeth was as follows: sound enamel: enamel of third molars free of caries, discolouration and any superficial defects; sound dentin: dentin of third molars free of caries, discolouration and any superficial defects; MIH-affected enamel: caries-free MIH affected first molars with white and yellow opacities having a diameter of at least 3 mm and no post eruptive breakdowns; dentin of MIH-affected first molars: caries-free specimens with post eruptive breakdowns and partially lost enamel. Teeth affected by other developmental or pathological conditions were excluded from the study. Selected teeth were stored in sealable containers of DDW until further use.

### 2.2. Specimen Preparation

Sectioning of the teeth commenced by removing the roots of the molars perpendicular to the long axis of the teeth using a 0.3 mm thick diamond wafering blade (IsoMet^TM^ Buehler, Lake Bluff, IL, USA). Subsequently, the crowns were sectioned in various planes as deemed necessary per individual tooth to provide the required specimens. Sound enamel specimens were obtained from the occlusal half of the tooth to avoid thinner enamel in the cervical half of the crown. Dentin samples were obtained by sectioning the crowns perpendicular to the occlusal plane in a mesio-distal plane and then horizontally at a distance of 1.2 mm (±0.2 mm) from the highest pulp horn. A maximum of four specimens were obtained per tooth.

Specimens were thereafter rinsed in DDW, patted dry, and placed with their flat surfaces against the base of cylindrical silicon moulds (Struers, Copenhagen, Denmark). A pourable mixture of cold-curing resin (Versocit-2 Struers, Copenhagen, Denmark) prepared according to the manufacturer’s instructions was carefully poured into the moulds and allowed to set. Once set, the embedded specimens were removed from their silicon moulds and manually polished with wet 600-grit carbide paper (Struers A/S, Copenhagen, Denmark) to produce evenly roughed flat surfaces of 5–6 mm.

### 2.3. Bonding and Pre-Treatment Protocols

Polished specimens were then rinsed with DDW, assigned a unique identification code, and randomised into 14 experimental groups using Microsoft Excel (Version 16.0, Microsoft Corp., Redmont, WA, USA). Control groups (N = 4) and test groups (N = 10) were bonded to sound teeth and MIH-affected teeth, respectively. Each experimental group consisted of 12 specimens (Table 1). Two adhesive systems, a universal adhesive in self-etch mode (Scotchbond^TM^ Universal Plus; Solventum, Kamen, Germany) (SBUP) and an etch-and-rinse adhesive (Adper ^TM^ Scotchbond 1XT; Solventum, Kamen, Germany) (ASA), were investigated in this study. The compositions of these adhesives are listed in Table 1. MIH-affected enamel specimens of six test groups were pre-treated with various agents prior to bonding with Scotchbond^TM^ Universal Plus. Bonding protocols for control and test groups 1 to 8 and pre-treatment and bonding protocols of test groups 9 to 14 are described in Table 2 and Table 3, respectively.

A custom-made hollow cylindrical plastic tube with an internal diameter of 2.3 mm and a height of 3 mm was placed on each specimen and firmly secured by the reciprocal crevice of a custom-made apparatus (Ultradent^TM^ Products, South Jordan, UT, USA). The plastic tube was packed with resin composite (Filtek^TM^ Universal Restorative, 3M ESPE, St. Paul, MN, USA) in three increments of 1mm each and individually light-cured for 10 s using an LED curing unit with a light intensity of 1470 mW/cm^2^ (−10%/+20%) (3M ESPE Elipar^TM^ Deep Cure-S, Solventum, Germany). Bonded specimens were stored in DDW and incubated at 37 °C for 24 h prior to shear bond strength testing.

### 2.4. Shear Bond Strength (SBS) Testing

Specimens were secured horizontally on a metal mounting jig, and they were loaded to failure at a crosshead speed of 1.0 mm/min using a customised stainless-steel shearing fixture that was attached to a universal testing machine (Type 1446 60 2010/TND Zwick Roell, Ulm, Germany). Shear bond strength values (MPa) were calculated as the peak loading force needed to shear the resin composite rods divided by the bonded surface area. The sheared resin composite rods we retrieved and examined to determine the mode of failure.

### 2.5. Modes of Failure

**Figure 1:**
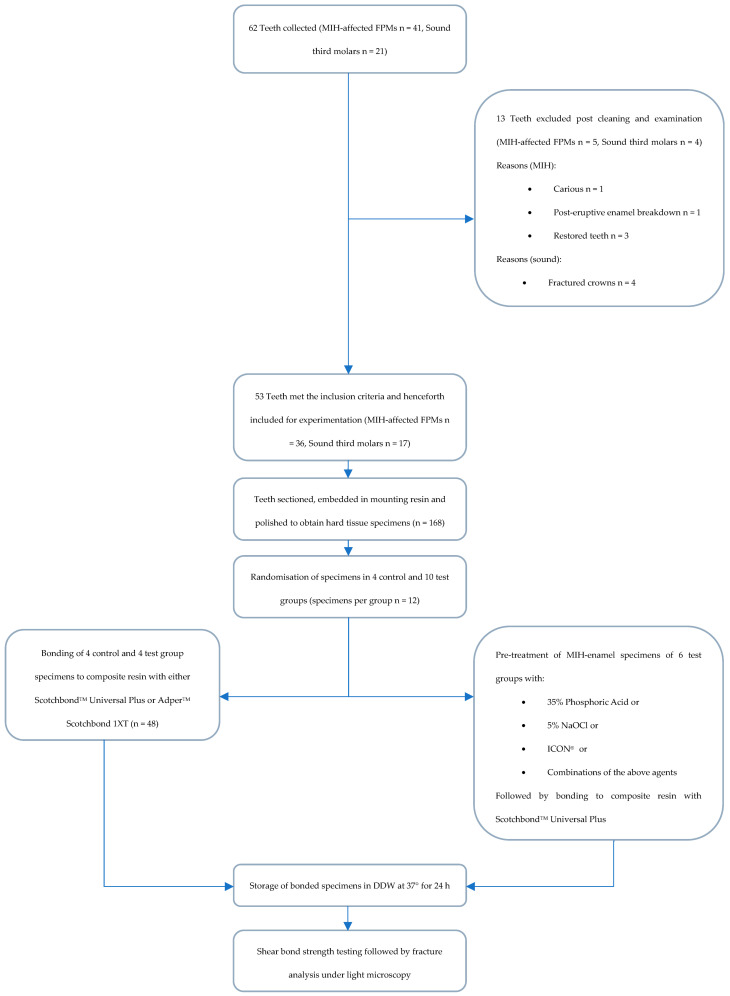
A summary of the methodology used in this study.

Following completion of SBS testing, debonded hard tissue surfaces and resin composite rods were examined under light microscopy at ×20 magnification. Failures were categorised as follows: adhesive failure at the hard tissue-resin adhesive interface, cohesive failure in composite, cohesive failure in hard tissue, or mixed failure that includes a partial cohesive and partial adhesive failure. Figure 1 summarises the methodology used in the study.

### 2.6. Statistical Analysis

All statistical tests were performed using SPSS software (version 26.0, IBM Corporation, New York, NY, USA), and level of significance was set at *p* ≤ 0.05 for all tests performed. Descriptive statistics were calculated for SBS values and modes of failure in each group. Normal distribution and equal variance of the SBS values were verified using the Shapiro–Wilk test (*p* ≤ 0.05). One-way analysis of variance (ANOVA) test and Bonferroni–Holm analysis were used to compare the mean SBS values amongst the 14 experimental groups and pairwise comparisons between these groups, respectively. The association between modes of failure in each group was examined with Pearson’s chi-square test.

## 3. Results

### 3.1. Shear Bond Strength Values

**Figure 2:**
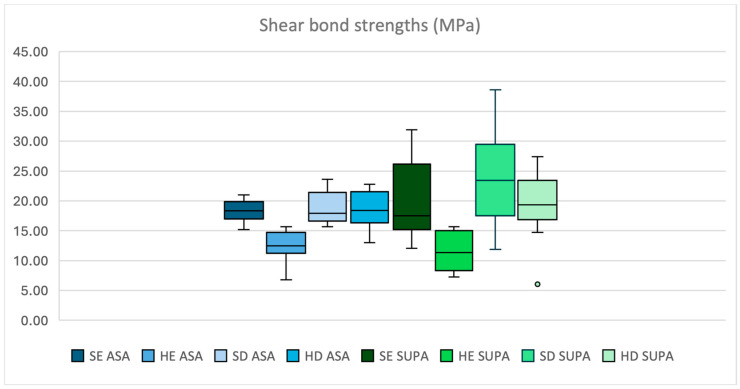
Overview of sound enamel (SE), hypomineralised enamel (HE), sound dentin (SD), and hypomineralised dentin (HD) results bonded using Scotchbond^TM^ Universal Plus (SUPA) and Adper^TM^ Scotchbond 1XT (ASA).

No pre-test failures were observed. The Shapiro–Wilk test revealed normal distribution for all groups except for group HE 2 (Scotchbond^TM^ Universal Plus) (*p* > 0.05). The mean SBS (MPa) values (±SD) are shown in Table 4. Amongst all 14 groups, the greatest mean SBS of resin composite was observed in sound dentin bonded with Scotchbond^TM^ Universal Plus adhesive (Group SD 2). The mean bond strength for sound enamel (Group SE 2) and hypomineralised enamel (Group HE 2) bonded with SBUP was significantly higher for sound enamel (19.68 ± 6.25 vs. 11.53 ± 3.29 MPa, *p* < 0.001), whereas the mean bond strengths for sound dentin did not differ significantly compared to those of MIH dentin for both adhesives (SBUP = 23.76 ± 7.68 vs. 19.49 ± 5.75 MPa, *p* > 0.05; ASA = 18.84 ± 2.71 vs. 18.50 ± 3.32 MPa, *p* > 0.05) (Figure 2).

**Figure 3:**
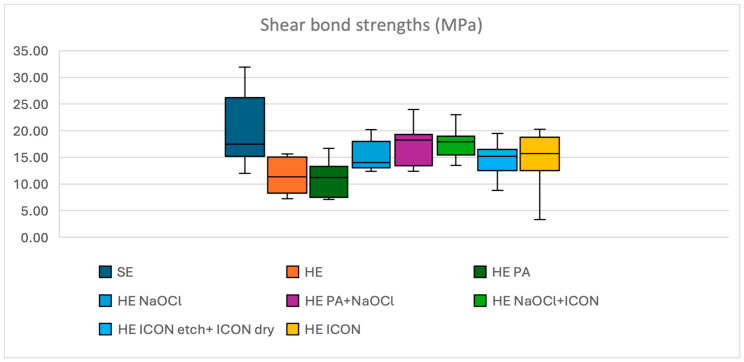
Overview of sound enamel (SE) and hypomineralised enamel (HE) results bonded using Scotchbond^TM^ Universal Plus. PA = phosphoric acid, NaOCl = sodium hypochlorite.

When compared to the mean shear bond strength to hypomineralised enamel for SBUP, acid etching using phosphoric acid prior to bonding in group HE 3 decreased the mean SBS value, whereas oxidative pre-treatment without and with acid etching in groups HE 4 and HE 5, respectively, and oxidative pre-treatment followed by resin infiltration in group HE 6 and pre-treatment with partial and complete resin infiltration in groups HE 7 and HE 8, respectively, increased the mean SBS values (Figure 3).

Overall, the mean SBS (MPa) values of the 14 groups differed significantly (one-way ANOVA, F ratio = 9.00, df = 167, *p* < 0.001). Bonferroni tests revealed significant differences in the mean SBS values between the groups and are depicted in Table 4.

### 3.2. Modes of Failure

The distributions of modes of failure within enamel and dentin groups are shown in Table 5 and Table 6, respectively. Overall, adhesive failures were the most common (75.0% of all failures), followed by mixed failures (17.8% of all failures). Failures in sound enamel (SBUP = 75.0%; ASA = 75.0%) and dentin (SBUP = 83.3%; ASA = 83.3%) were predominantly adhesive. From 168 specimens, only seven specimens (4.1%) showed cohesive failures in enamel, from which six failures (3.5%) involved hypomineralised enamel bonded with both adhesives, and one failure (0.5%) occurred in hypomineralised dentin bonded with Adper^TM^ Scotchbond 1XT. There was no association seen for the modes of failure between groups (χ^2^ = 47.93, *p* = 0.15).

## 4. Discussion

### 4.1. SBS to Sound Hard Tissue vs. Hypomineralised Tissue

The present study reported significantly higher mean SBS values for sound enamel (SE) compared with hypomineralised enamel (HE) for universal adhesive, Scotchbond^TM^ Universal Plus, used in self-etch mode. On the contrary, mean SBS values between SE and HE did not differ significantly for the etch-and-rinse adhesive, Adper^TM^ Scotchbond 1XT. Similarly, no significant differences were observed between the mean SBS values of sound dentin (SD) and hypomineralised dentin (HD) for both adhesives. Therefore, the first null hypothesis that “there is no difference in shear bond strengths to enamel and dentin of sound and MIH-affected molars” was rejected only for the differences in shear bond strengths to SE and HE bonded with universal adhesive Scotchbond^TM^ Universal Plus.

Published in vivo experiments have reported significantly higher bond strengths to SE compared to HE [18,22,24,25,32]. William et al., Chay et al., and Ekambaram et al. reported significantly higher mean MSBS values to SE compared to HE with adhesives Single Bond^TM^ and Clearfil^TM^ SE Bond [18], Clearfil^TM^ SE Bond [22] and Adper Single Bond 2, respectively [24]. Krämer et al. also reported significantly higher micro-tensile bond strengths to SE in comparison to HE with etch-and-rinse adhesives, Optibond FL, and Scotchbond^TM^ Universal and self-etch adhesive Clearfil^TM^ SE [25].

The inferior adhesion strength of resin composites to HE has been attributed to its inferior chemo-mechanical properties and haphazard, porous crystalline structure in comparison to sound enamel [33]. HE is associated with a 28% reduction in mineral content, 80% more carbonated apatite, and a 3- to 15-fold elevation in protein content. Qualitative evaluation of phosphoric acid etched HE using SEM has revealed poor intercrystal porosity and non-uniform dissolution of enamel prism cores and peripheral rods [18,22,24]. These characteristics of HE are responsible for deficient formation of resin tags, atypical etching patterns, and poor micromechanical retention—all of which play a crucial role in bonding to composite resin restorative materials whilst using dental adhesives [34]. As a result, altered chemo-mechanical properties of HE, as well as the excessively high content of carbonated apatite and proteins acting as a micro-mechanical and chemical barrier, contribute to the suboptimal bonding efficacy of HE [18,22,24,33].

The present study, however, reported no significant differences in the mean SBS of resin composite to SE compared to HE, bonded with an etch-and-rinse adhesive, Adper^TM^ Scotchbond 1XT. This may be attributed to the possibility of a greater number of HE specimens of mild severity, possessing mechanical and chemical properties similar to SE, being bonded with Adper^TM^ Scotchbond 1XT. Additionally, the residual water from rinsing prior to bonding with two-step etch-and-rinse dental adhesives has been postulated to interfere with the resin infiltration of etch-and-rinse dental adhesives [18].

To the authors’ knowledge, only Kraemer and colleagues have investigated and compared micro-tensile bond strengths of resin composite to sound dentin and dentin of MIH-affected teeth, bonded using Optibond FL, an etch-and-rinse adhesive, and Clearfil^TM^ SE Bond, a self-etch adhesive [25]. The authors of this experiment observed no significant differences in the mean micro-tensile bond strengths to sound and hypomineralised dentin bonded with Optibond FL. However, they did observe significantly higher mean bond strength values to SD as compared to HD, bonded with two-step self-etch adhesive Clearfil^TM^ SE. The present study reported no significant differences in the mean shear bond strengths to sound and hypomineralised dentin bonded with an etch-and-rinse adhesive and a self-etch adhesive. Heijs et al. investigated sound dentin and dentin beneath MIH-affected enamel and observed few morphological changes between the two. With the exception of interglobular dentin, they did not report any other structural changes and found that, in comparison with ameloblasts, odontoblasts of MIH-affected teeth were not affected [35]. This investigation underlines that SD and HD differ minimally in their morphology and chemical composition, which in turn may explain the non-significant results in the bond strengths of resin composite to SD and HD reported in the present study.

### 4.2. Pre-Treatment of Hypomineralised Hard Tissue

The integration of pre-treatment protocols to remove intrinsic proteins and fill porosities has been advocated with the aim of increasing the bond strengths of dental adhesives. Different types of pre-treatment methods prior to bonding with dental adhesives have reported mixed effects on the bond strength values of HE [22,24,26]. In the present study use of acid etching with 35% phosphoric acid prior to bonding with universal adhesive Scotchbond^TM^ Universal Plus decreased SBS values of HE. On the other hand, the use of oxidative pre-treatment followed by resin infiltration resulted in significantly higher mean SBS values compared to HE, with no significant differences in mean SBS values when compared to SE. This implies that pre-treatment with 5% NaOCl supplemented with ICON^®^ resin infiltration improved bond strengths to HE, confirming the results of Chay et al. [22].

Resin infiltration that uses a low-viscosity and highly penetrant resin was designed as a minimally invasive treatment modality with the purpose of penetrating deep into the enamel of non-cavitated carious lesions, and retard carious lesion progress [36]. It achieves this purpose by sealing porous channels and blocking the diffusion pathways for acid penetration and ionic movement in carious lesions [37]. The porosities in HE were thought to respond to resin infiltration similarly to the ones in caries defects and thereby provide an increased surface area for micromechanical retention of resin tags and possibly improve bonding to HE [22,33]. This form of pre-treatment has been documented in increasing shear bond strengths to HE as reported by Wiegand et al., who used hydrochloric acid etching rather than conventional phosphoric acid etching [38]. Nevertheless, the present study noted that treatment with resin infiltration prior to bonding with Scotchbond^TM^ Universal Plus did not significantly improve the SBS values to HE. Furthermore, Chay et al. reported the lowest MSBS values for pre-treatment with resin infiltration, performing worse than “routine” bonding to HE. The authors explained that the hydrophilic constituents of the increased protein content in HE act as a physical or chemical barrier that prevents adequate penetration of resin infiltrant, or the adhesive used in this experiment, Clearfil^TM^ SE Bond, did not bond to the resin infiltrant as predicted [22]. The present study adopted the modified resin infiltration procedure described by Chay et al. [22] and substituted 15% hydrochloric acid etching with 35% phosphoric acid etching as specimen preparation, with polishing paper that had removed the surface layer and thereby fulfilled the intent of the hydrochloric acid.

The present study also investigated the effects of partial resin infiltration with 15% hydrochloric acid (HCl) etching, followed by drying with ICON^®^ dry on SBS to HE. Surfaces of specimens belonging to this group were polished similarly to others to maintain uniform prepared surfaces throughout all specimens. To compensate for the double surface layer removal during specimen polishing and once again during acid etching, the working time of HCl was reduced to 1 min from the recommended etching time of 2 min. The recorded mean SBS values of HE using partial resin infiltration did not significantly differ from those of HE pre-treated using complete ICON^®^ resin filtration and HE without pre-treatment. This may be attributed to the intrinsic proteins in HE, which could not be deproteinised by the application of HCl and ICON^®^ dry, thus acting as barriers to the penetration of adhesive resin. Additionally, the more viscous dental adhesive might not have been able to penetrate the etched and dried hypomineralised tissue to a depth that would have been achievable by the less viscous resin infiltrant.

Removal of proteins in HE with deproteinising agents such as 5% NaOCl has yielded mixed results in past investigations. In the present study, oxidative pre-treatment with 5% NaOCl post acid etching did not improve bond strengths to HE, as was the case with oxidative pre-treatment followed by resin infiltration. Chay et al. reported that oxidative pre-treatment of HE with 5.25% NaOCl with and without ICON^®^ resin infiltration significantly increased bond strengths to HE, irrespective of the severity of hypomineralisation (creamy white or yellow-brown lesions) [22]. Ekambaram et al. also reported significantly increased bond strengths to MIH-affected enamel following pre-treatment with 5% NaOCl post acid etching [24]. However, they noted that deproteinisation significantly improved bond strength to HE of creamy white specimens and not of yellow-brown specimens. On the contrary, Krämer et al. reported that oxidative pre-treatment with 5.25% NaOCl alone and in combination with ICON^®^ resin infiltration had no significant impact on the microtensile bond strengths to HE, but instead caused fewer pre-test failures compared to HE that was not pre-treated [25]. Furthermore, the effects of oxidative pre-treatment with (post oxidative treatment) and without 35% phosphoric acid etching were compared, and no differences in bond strengths to HE were recorded for both groups. Likewise, Gandhi et al. investigated the effects of oxidative pre-treatment on bond strengths of resin sealant to HE with the use (post pre-treatment) and non-use of acid etching, and recorded no marked differences between the two methods [26].

To conclude, the present study reported that pre-treatment with 5% NaOCl followed by ICON^®^ resin infiltration significantly increased bond strengths to HE. Hence, the second null hypothesis that “various pre-treatment protocols of HE do not influence adhesion to MIH enamel” was rejected only for the pre-treatment protocol of oxidative pre-treatment with 5% NaOCl followed by ICON^®^ resin infiltration.

### 4.3. Adhesion to Hypomineralised Tissue Bonded Using Two Different Adhesives

Since the introduction of universal dental adhesives in 2010, they have been described as contemporary novelties possessing an unparalleled complex composition that simultaneously promotes the demineralisation of mineralised tissue, infiltration of resin monomers, and resin polymerization [39,40,41,42,43]. The matrix of these adhesives is based on an intelligent bond of hydrophilic and hydrophobic monomers, which allows concomitant bonding to both hydrophilic dental substrate and hydrophobic resin-based restorative material under various surface moisture conditions [43,44]. This versatile property of universal adhesives, along with the knowledge that HE like sound dentin contains increased water, makes their application to HE desirable. Therefore, a universal adhesive in self-etch mode was investigated in the present study. Bond tests revealed no significant differences in bond strengths to sound and hypomineralised enamel and dentin bonded with a universal adhesive in self-etch mode and an etch-and-rinse adhesive. Hence, the third hypothesis stating “there is no difference in shear bond strengths of resin composite between self-etch universal adhesive and etch-and-rinse adhesive” was not rejected.

Published in vitro experiments on adhesion strengths of various dental adhesive systems used in different modes and application methods have demonstrated mixed results [18,25,32]. William et. al. reported no marked differences between the mean bond strength values of a two-step self-etch adhesive, Clearfil^TM^ SE Bond, and an etch-and-rinse adhesive, 3M Single Bond [18]. On the other hand, Krämer et al. observed the lowest microtensile bond strength values in HE for the etch-and-rinse adhesive Scotchbond^TM^ Universal and the two-step self-etch adhesive Clearfil^TM^ SE Bond. In the same study, another etch-and-rinse adhesive, OptiBond^TM^ FL, yielded significantly higher mean microtensile bond strengths to hypomineralised enamel and dentin compared to Clearfil^TM^ SE Bond. This study reported the maximum number of pre-test failures for Clearfil^TM^ SE Bond [25]. Lee et al. investigated the adhesive strengths of universal adhesive Scotchbond^TM^ Universal in self-etch and etch-and-rinse modes to sound and hypomineralised enamel and found significantly higher mean MSBS for the universal adhesive applied in etch-and-rinse mode [32].

William et al. proposed two factors that might promote the bonding of self-etch adhesives to hypomineralised enamel: (1) the omission of rinsing and, along with it, the elimination of the interface of residual water on the bond, and (2) the micromechanical and chemical bonding of self-etch adhesives to hydroxyapatite. These authors also commented on the limited adhesion of etch-and-rinse adhesives to hypomineralised enamel and attributed this effect to the inadequate formation of micro-tags, consequential to the formation of little intercrystal porosity and the extrinsic moisture from rinsing after acid etching, which inhibits resin infiltration, resulting in a lowering of bond strengths. Lygidakis et al. reported that acetone found in some etch-and-rinse adhesives can eliminate residual water from rinsing post-etching, making enamel more available to bond [45].

### 4.4. Strengths and Limitations

While this investigation provides valuable insights into potential bonding strategies to improve bond strengths of composite resin to MIH-affected hard tissues by comparing two adhesive systems and the performances of various pre-treatments, the relatively low sample size per experimental group may have limited statistical power. As a result, subtle differences between the tested adhesive systems and the multiple pre-treatments may not have been revealed. The reason for a small sample size in the present study was the difficulty in obtaining MIH-affected FPMs that fulfilled the study’s inclusion criteria. Most of MIH teeth indicated for extraction were either carious and/or restored and/or exhibited post-enamel-breakdown. Future investigations should conduct a power analysis prior to investigating larger sample sizes to allow for detection of subtler differences amongst multiple experimental groups.

Another limitation of this study is represented by the heterogeneity of the hypomineralised specimens. Literature has reported that mechanical and chemical properties of less severe or creamy white hypomineralised lesions differ from those of severely affected or yellow-brown lesions, which in turn affect bond adhesion to HE [24,46]. An attempt to reduce the variability arising from the heterogeneity of the specimens was made by randomising the allocation of specimens into experimental groups. While randomisation minimises selection bias and strives for even distribution of the variability across the experimental groups, it does not ensure elimination of biological variability which in turn can impact shear bond strengths and the interpretability of group comparisons. Future investigations of treatment interventions on MIH-hard tissues should consider stratification of specimens based on lesion severity whilst designing their studies to eliminate differences in adhesive performance that are substrate dependent.

Another key limitation of this study is the absence of a scanning electron microscopy (SEM) analysis of the sheared hard tissue interfaces. High-resolution images generated by SEM would have enabled a valuable qualitative analysis of the microstructural characteristics of sound and MIH-affected hard tissues and hard tissue-composite interfaces [47]. Additionally, the analysis would have delivered a visual understanding of the microscopic key features of adhesion, such as etching patterns and resin tag penetration along with morphological alterations of MIH-affected enamel surfaces following different pre-treatment protocols [22,48]. Moreover, it would have aided in visualising and comprehending the effects of variables such as hard tissue substrates, dental adhesives and pre-treatment protocols on these key features. Whilst shear bond strength analysis is an integral part of composite-dental hard tissue adhesion investigations, SEM analysis helps to draw definite conclusions about the mechanisms underlying the bond strength values obtained, particularly in relation to how the adhesives and pre-treatments interact with the sound and structurally compromised MIH substrates and hence aids in deriving clinical recommendation of the investigated materials [47]. It is therefore the recommendation of the authors of this study that a qualitative analysis of the adhesion interfaces post shear testing be integrated in future experiments investigating bond strengths to dental hard tissues.

Furthermore, the investigation of universal adhesive Scotchbond^TM^ Universal was limited to its performance as a self-etch adhesive. This represents another limitation of the present study. While this investigation aimed at comparing two commonly used adhesive systems representing two different bonding strategies, future researchers testing universal adhesives in bond strength studies should investigate its performance in both self-etch and etch-and-rinse modes, as this will allow for direct comparison of the performances of both modes while having the advantage of a single adhesive composition.

The present study investigated shear bond strengths of composite resin to hypomineralised hard tissues bonded by two different adhesives in two different modes of application and a wide range of pre-treatments. The sound hard tissue substrates used in this study were obtained from sound teeth and not from unaffected areas of MIH-affected teeth as enamel that is apparently unaffected by hypomineralisation has been reported to show changes in its structure when compared with sound enamel [20,49], thus compromising adhesion of restorative materials even after extensive removal of dental tissue [50]. Additionally, this study also tested dentinal hard tissue from MIH-affected teeth and is, to the authors’ best knowledge, one of two studies to investigate bond strengths to MIH-affected dentin.

### 4.5. Clinical Relevance

The present study addresses a significant challenge in restorative dentistry: achieving reliable adhesion to MIH-affected hypomineralised enamel and dentin. The structurally and chemically compromised composition of hypomineralised hard tissues compounded by factors such as hypersensitivity and post-eruptive breakdown contribute to increased failure rates of composite restorations in MIH-affected teeth. By comparing bond strengths of a universal adhesive in self-etch-mode and a conventional etch-and-rinse adhesive, this study provides valuable insights into which adhesive strategy may offer more predictable outcomes in compromised hard tissues of MIH-affected teeth. Additionally, the study investigated various pre-treatment protocols to assess whether their application can improve bond strengths in hypomineralised enamel and successfully demonstrated that a minimally invasive technique advocating partial rather than complete removal of hypomineralised enamel followed by pre-treatment with NaOCl and subsequent resin infiltration may be considered a viable treatment option for MIH-affected enamel. These results support the non-invasive treatment of intact hypomineralised enamel and dentin lesions and contribute to the evidence on adhesion strategies in structurally compromised, hypomineralised hard tissues.

While the application of this pre-treatment protocol may pose fewer challenges when performed in the confines of a laboratory on extracted specimens, its use in clinical practice needs to be investigated. Patient cooperation, preventive measures for moisture control (e.g., dental damn), as well as increased treatment time and higher treatment costs are factors to be considered while contemplating this treatment option. The associated hurdles in its clinical translation make its use in young school-aged children, uncooperative patients, and in settings with limited resources related to armamentaria, personnel, and finances more challenging.

### 4.6. Future Prospect of the Present Study

Data on MIH prevalence reports its presence in more than 50 countries, making it a global burden [51]. As challenges associated with the management of MIH-affected teeth increase, so does the requirement for well-founded research which would aim at solving these challenges. The present study contributes to tackling these challenges by adding to the evidence of literature on adhesive strategies to hypomineralised hard tissues. Additionally, the outcome of this investigation can be used for the development of clinically effective adhesive protocols specifically designed for the restorative management of MIH-affected teeth with composite resin. Furthermore, this research contributes to evidence-based recommendations that could guide clinicians in opting for the most suitable treatment options in the restorative management of MIH-affected teeth. Lastly, these findings could influence the future formulations of MIH-targeted dental adhesive and pre-treatment agents and their application protocols with the aim of reducing restorative failures, increasing longevity of composite restorations and as a result positively impacting the life quality of MIH patients.

## 5. Conclusions

The following conclusions can be drawn from the present study:

1. Hypomineralised enamel bonded with a universal adhesive showed inferior adhesion compared to sound enamel;
2. Oxidative pre-treatment with 5% NaOCl followed by resin infiltration enhanced bond strength of composite resin to hypomineralised enamel;
3. Both dental adhesives, Scotchbond^TM^ Universal Plus and Adper^TM^ Scotchbond 1XT can be used for bonding to hypomineralised hard tissues;
4. Hypomineralised enamel specimens were associated with a higher number of cohesive failures.

## Figures and Tables

**Table 1:** Composition of materials used in the study.

Material	Constituents	Type	pH	Manufacturer
Scotchbond^TM^ Universal Plus Adhesive	MDP Phosphate Monomer, HEMA, Vitrebond copolymer, filler, ethanol, water, initiators, silane, dual-cure accelerator, dimethacrylate resins containing a BPA derivative-free, crosslinking radiopaque monomer	One-step self-etch	2.7	3M Deutschland GmbH, Neuss, Germany (now: Solventum, Kamen, Germany)
Adper^TM^ Scotchbond 1XT Adhesive (Etchant: Scotchbond^TM^ Universal Etchant)	Adhesive: BISGMA, HEMA, UDMA, ethanol, water, photoinitiator system, 1,3-dimethacrylate, copolymer of polyacrylic and itaconic acids, N,n-dimethylbenzocaine Etchant: 32% phosphoric acid, water	Two-step etch-and-rinse	4.7 <1	3M ESPE Deutschland GmbH, Neuss, Germany (now: Solventum, Kamen, Germany)
Filtek^TM^ Universal Restorative	AUDMA, AFM, di-urethane-DMA,1,12-dodecane-DMA, non-aggregated 4–11 nm zirconia filler, aggregated zirconia/silica cluster filler (20 nm silica and 4–11 nm zirconia particles), ytterbium trifluoride filler of agglomerated 100 nm particles.	Visible-light-activated restorative composite with nanofillers	-	3M, ESPE Dental Products, St. Paul, MN, USA
Verso Cit-2	Powder: dibenzoyl peroxide, methyl methacrylate Liquid: tetrahydrofurfuryl methacrylate, methacrylic acid, monoester with propane-1,2-diol tetramethylene dimethacrylate N, N-dimethyl-p-toluidine	Two-component acrylic mounting system	-	Struers, Ballerup, Denmark
Scotchbond^TM^ Etchant	35% phosphoric acid, water, Poly (vinyl alcohol)	Gel	~1	3M Deutschland GmbH, Neuss, Germany (now: Solventum, Kamen, Germany)
Histolith NaOCl	5% sodium hypochlorite	-	11.8	Lege artis Pharma GmbH, Dettenhausen, Germany
ICON^®^	Etchant: 15% HCL, pyrogenic silicic acid Drying Agent: 99% Ethanol, Infiltrant: Methacrylate-based resin matrix, Initiators, additives	-	-	DMG, Hamburg, Germany

**Table 2:** Bonding protocols for control and test groups 1 to 8.

Group	Tooth Tissue	Adhesive	Etching	Conditioning	Composite Packing and Polymerisation
SE 1	Enamel, unaffected	Adper^TM^ Scotchbond 1XT (etch-and-rinse)	32% phosphoric acid Etch (15 s), Water spray (10 s), Blotting excess moisture using a cotton pellet	Double player of adhesive applied (15 s), Air-thinned (5 s), Polymerisation (10 s)	Composite resin packed in 3 increments of 1 mm, Polymerisation (10 s per increment)
HE 1	Enamel, affected				
SD 1	Dentin, unaffected				
HD 1	Dentin, affected				
SE 2	Enamel, unaffected	Scotchbond^TM^ Universal Plus (self-etch)	-	Single layer of adhesive applied in a rubbing motion (20 s), Air thinned (5 s), Polymerisation (10 s)	Composite resin packed in 3 increments of 1 mm, Polymerisation (10 s per increment)
HE 2	Enamel, affected				
SD 2	Dentin, unaffected				
HD 2	Dentin, affected				

Control groups (SE = sound enamel, SD = sound dentin), Test groups (HE = hypomineralised enamel, HD = hypomineralised dentin).

**Table 3:** Pre-treatment and bonding protocols for test groups 9 to 14.

Scothcbond^TM^ Universal Plus (Self-Etch Mode)						
**Group**	HE 3	HE 4	HE 5	HE 6	HE 7	HE 8
**Pre-** **treatment** **Agents**	35% PA	5% NaOCl, ICON^®^ dry	35% PA, 5% NaOCl, ICON^®^ dry	35% PA, 5% NaOCl, ICON^®^ dry, ICON^®^ infiltrant	ICON^®^ etch, ICON^®^ dry	35% PA, ICON^®^ dry, ICON^®^ infiltrant
**Acid Etching**	35% PA (15 s)	-	35% PA (15 s)	35% PA (15 s)	ICON^®^ etch (60 s)	35% PA, (15 s)
**Rinsing**	Water spray (15 s)	-	Water spray (15 s)	Water spray (15 s)	Water spray (30 s)	Water spray (15 s)
**Pre-** **treatment**	Air dried (30 s)	5% NaOCl applied with microbrush in back and forth rubbing motion (1 min) Water spray (30 s) ICON^®^ dry (30 s) Air dried (30 s)	5% NaOCl applied with microbrush in back and forth rubbing motion (1 min) Water spray (30 s) ICON^®^ dry (30 s) Air dried (30 s)	5% NaOCl applied with microbrush in back and forth rubbing motion (1 min) Water spray (30 s) ICON^®^ dry (30 s) Air dried (30 s)	ICON^®^ dry (30 s) Air dried (30 s)	ICON^®^ dry (30 s) Air dried (30 s)
**Resin** **infiltration**	-	-	-	ICON- infiltrant (3 min) Excess removed with cotton roll and light-cured (40 s)	-	ICON- infiltrant (3 min) Excess removed with cotton roll and light-cured (40 s)
**Conditioning**	1 coat Scotchbond^TM^ Universal Plus in rubbing motion (20 s), Air thinned, (5 s) Polymerisation (10 s)	1 coat Scotchbond^TM^ Universal Plus in rubbing motion (20 s), Air thinned, (5 s) Polymerisation (10 s)	1 coat Scotchbond^TM^ Universal Plus in rubbing motion (20 s), Air thinned (5 s), Polymerisation (10 s)	1 coat Scotchbond^TM^ Universal Plus in rubbing motion (20 s), Air thinned (5 s), Polymerisation (10 s)	1 coat Scotchbond^TM^ Universal Plus in rubbing motion (20 s), Air thinned (5 s), Polymerisation (10s)	1 coat Scotchbond^TM^ Universal Plus in rubbing motion (20 s), Air thinned (5 s), Polymerisation (10s)

HE = hypomineralised enamel, PA = phosphoric acid, NaOCl = sodium hypochlorite.

**Table 4:** Mean SBS values (SD) in sound and hypomineralised enamel and dentin. Values with the same superscript alphabet indicate a significant difference at α ≤ 0.05 (one-way ANOVA test).

Adhesive	Group	Shear Bond Strength (SD) (MPa)
Adper^TM^ Scotchbond 1XT	SE 1	18.19 (1.83) ^a,b^
	HE 1	12.56 (2.44) ^c,d,e,f,I,s,v,y^
	SD 1	18.84 (2.71) ^f,g,h,i^
	HD 1	18.50 (3.32) ^j,k^
Scotchbond^TM^ Universal Plus	SD 2	23.76 (7.68) ^d,l,m,n,o,p,q,r,s^
	HD 2	19.49 (5.75) ^e,t,u,v^
	SE 2	19.68 (6.25) ^c,w,x,y^
	HE 2	11.53 (3.29) ^a,g,j,l,t,w,z^
	HE 3	10.73 (3.20) ^b,h,k,m,u,x,β,δ^
	HE 4	15.27 (2.72) ^n^
	HE 5	17.23 (3.72) ^o,β^
	HE 6	17.84 (2.98) ^p,z,δ^
	HE 7	14.40 (3.17) ^q^
	HE 8	14.82 (4.62) ^r^

Control groups (SE = sound enamel, SD = sound dentin), Test groups (HE = hypomineralised enamel, HD = hypomineralised dentin).

**Table 5:** Results of modes of failure for all enamel groups (fracture type, N [%]): adhesive fracture (AF), cohesive fracture enamel (CFe), cohesive fracture composite (CFc), mixed fracture (MF).

Adhesive	Group	N	AF	CFe	CFc	MF
Adper^TM^ Scotchbond 1XT	SE 1	12	9 [75]	0 [0]	0 [0]	3 [25]
	HE 1	12	6 [50]	3 [25]	0 [0]	3 [25]
Scotcbond^TM^ Universal Plus	SE 2	12	9 [75]	0 [0]	0 [0]	3 [25]
	HE 2	12	9 [75]	2 [17]	0 [0]	1 [08]
	HE 3	12	10 [83]	0 [0]	0 [0]	2 [17]
	HE 4	12	10 [83]	0 [0]	0 [0]	2 [17]
	HE 5	12	7 [58]	0 [0]	0 [0]	5 [42]
	HE 6	12	6 [50]	2 [17]	0 [0]	4 [33]
	HE 7	12	10 [83]	0 [0]	0 [0]	2 [17]
	HE 8	12	10 [83]	0 [0]	0 [0]	2 [17]

SE = sound enamel, HE = hypomineralised enamel.

**Table 6:** Results of mode of failure for all dentin groups (fracture type, N [%]): adhesive fracture (AF), cohesive fracture enamel (CFe), cohesive fracture composite (CFc), mixed fracture (MF).

Adhesive	Group	N	AF	CFe	CFc	MF
Adper^TM^ Scotchbond 1XT	SD 1	12	10 [83]	0 [0]	0 [0]	2 [17]
	HD 1	12	10 [83]	0 [0]	1 [08]	1 [08]
Scotcbond^TM^ Universal Plus	SD 2	12	10 [83]	0 [0]	1 [08]	1 [08]
	HD 2	12	10 [83]	1 [08]	1 [08]	0 [0]

SD = sound dentin, HD = hypomineralised dentin.

## Data Availability

The raw data supporting the conclusions of this article will be made available by the authors on request.
